# Nothing so practical as theory: a rapid review of the use of behaviour change theory in family planning interventions involving men and boys

**DOI:** 10.1186/s12978-021-01173-0

**Published:** 2021-06-13

**Authors:** Martin Robinson, Áine Aventin, Jennifer Hanratty, Eimear Ruane-McAteer, Mark Tomlinson, Mike Clarke, Friday Okonofua, Maria Lohan

**Affiliations:** 1 School of Nursing and Midwifery and Centre for Evidence and Social Innovation, Queen’s University Belfast, Belfast, Northern Ireland UK; 2 School of Public Health, University College Cork, Cork, Ireland; 3 Institute for Life Course Health Research, Stellenbosch University, Stellenbosch, South Africa; 4 Women’s Health Action Research Centre, Benin City, Edo State Nigeria

**Keywords:** Review, Theory of change, Behaviour change theory, Interventions, Family planning

## Abstract

**Background:**

There is growing recognition of the need for interventions that effectively involve men and boys to promote family planning behaviours. Evidence suggests that the most effective behavioural interventions in this field are founded on theoretical principles of behaviour change and gender equality. However, there are few evidence syntheses on how theoretical approaches are applied in this context that might guide best practice in intervention development. This review addresses this gap by examining the application and reporting of theories of behaviour change used by family planning interventions involving men and boys.

**Methods:**

We adopted a systematic rapid review approach, scoping findings of a previously reported evidence and gap map of intervention reviews (covering 2007–2018) and supplementing this with searches of academic databases and grey literature for reviews and additional studies published between 2007 and 2020. Studies were eligible for inclusion if their title, abstract or keywords referred to a psychosocial or behavioural intervention targeting family planning behaviours, involved males in delivery, and detailed their use of an intervention theory of change.

**Results:**

From 941 non-duplicate records identified, 63 were eligible for inclusion. Most records referenced interventions taking place in low- and middle-income countries (65%). There was a range of intervention theories of change reported, typically targeting individual-level behaviours and sometimes comprising several behaviour change theories and strategies. The most commonly identified theories were Social Cognitive Theory, Social Learning Theory, the Theory of Planned Behaviour, and the Information-Motivation-Behaviour Skills (IMB) Model. A minority of records explicitly detailed gender-informed elements within their theory of change.

**Conclusion:**

Our findings highlight the range of prevailing theories of change used for family planning interventions involving men and boys, and the considerable variability in their reporting. Programmers and policy makers would be best served by unified reporting and testing of intervention theories of change. There remains a need for consistent reporting of these to better understand how complex interventions that seek to involve men and boys in family planning may lead to behaviour change.

## Plain language summary

Family planning (FP) programmes aim to enable people to achieve their desired family size. Successful programmes are essential for encouraging better health outcomes for individuals and families. Historically, FP programmes have focused on the sexual and reproductive health and rights of women and girls, and while this is necessary, it has also contributed to the exclusion of men and boys from FP programmes. There is growing evidence to suggest, however, that involving men and boys in FP may increase its uptake and improve health outcomes for all. However, we still know relatively little about the best ways to involve men and boys in FP programmes in order to ensure success. Research is ongoing to rectify this.

When we look at public health behaviour change programmes in general, we find that many successful programmes incorporate theories of behaviour change into their design. These theories guide the kinds of activities and materials that the programme employs in order to promote behaviour change. In the field of FP, there is little information about the kinds of behaviour change theories that might be used to design programmes. This review aimed to address this by reviewing the global literature on FP programmes that involved men and boys to identify relevant behaviour-change theories. We found a range of theories that will be of use to programme planners. We also found, however, that there was lots of variability in the way theories were reported. We make recommendations for how this problem might be resolved.

## Background

Family planning (FP) interventions aim to provide information and skills to enable individuals to achieve their desired family size and effectively plan the timing of births. This is essential to achieving reproductive health and rights for women and families [1]. Ensuring effective FP and uptake of FP interventions is a public health concern, not least in low- and middle-income countries (LMICs) where unmet need for family planning is high. It is argued that the promotion of FP and Sexual and Reproductive Health and Rights (SRHR) together is central to advancing individual wellbeing and to socioeconomic development [2].

The 1994 International Conference on Population and Development called for greater male involvement in FP and SRHR [3]. Since then, programmers and national strategies have sought to do this by widening provision, tailoring and adapting programmes, and encouraging male participation. Despite this progress, it remains that the role of men is often relegated to that of supporting their female partners in FP decision-making, rather than also being active users of FP methods themselves [4]. Men and boys are still underserved and under-involved in FP programming even though there is increasing evidence that they can play a key role in FP intervention effectiveness, increasing uptake of FP, and enabling maternal SRHR [4, 5].

A key part of effective design in FP intervention is the application of a theory of behaviour change to guide development [6]. A ‘theory of change’ is a mechanistic description of causal determinants, in other words, a theoretical depiction of how an intervention is intended to lead to change in a specified outcome [6]. Generally, theories of change are based on established behaviour change theories and they usually detail how programme components are expected to lead to change. Theories of change may encompass psychosocial determinants of behaviour, positing how environmental and programme inputs lead to output behaviours via individual, interpersonal, and structural processes. Well known examples of this are the Theory of Planned Behaviour [7] and Social Learning Theory [8]. Such theories provide a framework to understand how influencing knowledge, skills, attitudes, and environmental factors may lead to the desired behaviour change. In the field of SRHR, gender norms and masculinities are likewise recognised as important theoretical factors influencing a wide range of SRHR behaviours and outcomes [9]. Reflecting this, there have been calls from policy makers and statutory agencies to better integrate gender norms in interventions in contemporary SRHR strategies [10, 11]. Despite these calls and tentative indications of positive effects, evidence suggests that the application of gender-transformative principles and strategies is not yet widely applied [12, 13].

Notwithstanding the importance of effective and evidence-based FP intervention, the theoretical grounding and processes of complex interventions relating to FP remain underinvestigated in systematic reviews [14]. There is a lack of cohesive literature on the commonly applied frameworks within interventions in this area, particularly those involving men and boys. A previously conducted review of theory-based interventions found that Social Cognitive Theory was the most frequently used in interventions to promote contraceptive use, often in conjunction with another model of behaviour change [14, 15]. That review, and others in the field, however, focus largely on female use of contraceptives or do not parse interventions based on participant gender or sex. The review reported here addresses this gap, asking a more specific research question that will provide pragmatic information for those wishing to develop interventions that effectively involve men and boys in FP: What theories of change have been used to inform FP interventions involving with men and boys?

## Methods

### Design

This paper presents a synthesis based on a Rapid Review, an approach to data acquisition that employs a systematic but restricted approach to the capture and analysis of literature [16]. Most commonly, rapid reviews are limited in their methods and scope to aid more timely synthesis for instance by searching only peer-reviewed literature and extracting only very specific information from studies [17]. Initial evidence suggests that their results largely coincide with those of full systematic reviews of the same topic while offering more timely completion [16].

Systematic evidence reviews typically focus on examining the outcome effectiveness of interventions [18]. A contrasting approach to this is that of a Realist Review. This approach attempts to synthesise the theoretical and empirical evidence to understand “what works for whom, in what circumstances, in what respects and how” [18]. As the goal of this review was to identify the context and use of theories of change, the data analysis and synthesis draws on the Realist approach.

More information on the review design and methodology is available in the review protocol [19]. This rapid review was conducted as part of an ongoing systematic review that aims to identify the effective components and characteristics of interventions involving men and boys in LMICs in family planning [20].

### Inclusion and exclusion criteria

Records were eligible for inclusion if their title, abstract or keywords referred to FP intervention(s), targeted psychosocial or behaviour FP outcomes, mentioned use of a theory of change, and involved males in the intervention. As this review aimed to obtain a broad overview of the use of theories of change no limits were applied in terms of study design, therefore, intervention design papers, evaluations, protocols, and reviews were all eligible for inclusion. Similarly, eligible interventions were limited only to psycho-social or behavioural designs encouraging capacity or engagement with FP. Records related exclusively to biomedical interventions and outcomes (e.g. evaluating surgical procedures, investigating fertility rate following vasectomy) were not within the scope of this review and therefore excluded.

### Search strategy

We identified studies in two ways. First, we screened all the reviews included within a comprehensive Evidence and Gap Map (EGM) and systematic review of reviews which the authors were involved in and is freely available online [12]. The EGM contained reviews interventions reporting a range of SRHR outcomes for men and boys published between 2007 and 2018 [12]. Second, we searched academic databases and grey literature sources for reviews, articles, and protocol documents published between 01 January 2007 and 05 May 2020 (the date of searches). Searches for academic literature were limited to title, abstract and keywords, and conducted using the following databases: PubMed, CINAHL, PsychINFO, and the Cochrane Library (including CENTRAL). Potentially relevant grey literature was identified using abridged versions of the search terms used for the academic searches in Google and Google Scholar. After sorting by relevance, the first five pages of records returned in each grey literature source were screened.

Search terms were prepared based on those used by the previously cited EGM [12, 21] and adapted according to the specific goals of this review. We combined terms for FP, men and boys, intervention and theory using the AND operator, see Appendix 1 for full list of terms.

Record screening was carried out by one author (MR) by title only to remove obviously irrelevant records e.g. those that clearly did not relate to a psychosocial intervention, involve males, or did not describe a theory of behaviour change. A random sample comprising 10% of excluded records was checked by a second author (ÁA) for quality control. Restricting verification of a subset of records is recognised in Rapid Review methodology [16, 22]. While full dual screening is preferable, guidelines indicate screening training using a limited set of records as low as 10–20% may be sufficient to ensure consistent decisions are made [23].

The first 100 titles and abstracts were double screened independently by two authors (MR and ÁA) against our inclusion criteria [19]. Studies were excluded if they related only to a biomedical FP intervention or outcomes (e.g. sperm viability after biomedical intervention). Any disagreement was resolved through discussion and this double screening was repeated a second time, at which point we were satisfied that it was sufficient for one author to single screen all remaining records. A random sample of 10% of included records were likewise checked by a second reviewer (ÁA) to maintain screening quality.

Full text screening and data extraction of the first five records was conducted by two authors (MR and ÁA) independently. Disagreements were once again resolved through discussion until authors were satisfied that one author (MR) could complete the remaining data extraction alone.

### Search procedure

Reviews within the EGM were considered independent records for the purposes of this review. Review of titles and abstracts of items in the EGM showed that most of the included 145 records related to FP programmes were medical interventions (*n* = 79) and therefore not relevant for the present review. The remaining 66 full text records were screened to determine if they included details of intervention theory of change. In total 46 (70%) of these systematic reviews did not detail the use of behaviour change theory and were excluded, the remaining 20 systematic reviews (30%) were included in the review.

Of the 796 non-duplicate records identified from our separate academic database and grey literature search, 666 were excluded based on title and abstract screening. The remaining 130 records (*n* = 104 academic database records and *n* = 26 grey literature records) were subject to full text screening. Of these, ten were excluded because they did not report a behavioural or psychosocial intervention, 37 were excluded for lacking behavioural FP outcomes, 13 did not report male involvement, and 27 did not report detail on the theory(ies) of change applied. This left 43 records from the academic and grey literature search to be included in the review. Combined with the 20 reviews identified from the EGM, this meant a total of 63 records were included in analysis and synthesis (see Appendix 2).

### Data extraction and analysis

Information regarding the characteristics and intended outcomes of interventions was extracted from records in addition to any reference to a published behaviour change theory or programme-specific theory of change. Where records (e.g. systematic reviews) contained information on multiple interventions, this information was extracted where available for all relevant interventions. Finally, brief details on how the theory of change was applied in practice was extracted from each record to aid synthesis.

Given the heterogeneity of included records and the objectives of this review, the data were analysed using narrative synthesis methods [24] and informed by a realist approach [25]. This involved adopting a flexible approach to data synthesis and reporting to articulate the design of interventions, and the intended mechanisms of theory in their design [25]. Drawing on these approaches the sum evidence for each study was grouped thematically in relation to intended outcomes, context, intervention design, and elements of theoretical underpinning [24, 25].

## Results

**Fig. 1:**
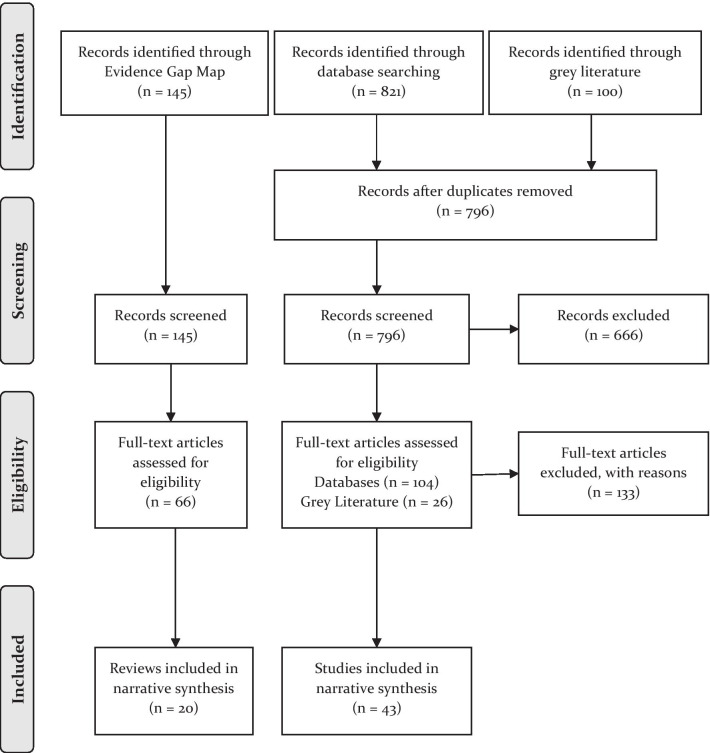
PRISMA study flow

**Fig. 2:**
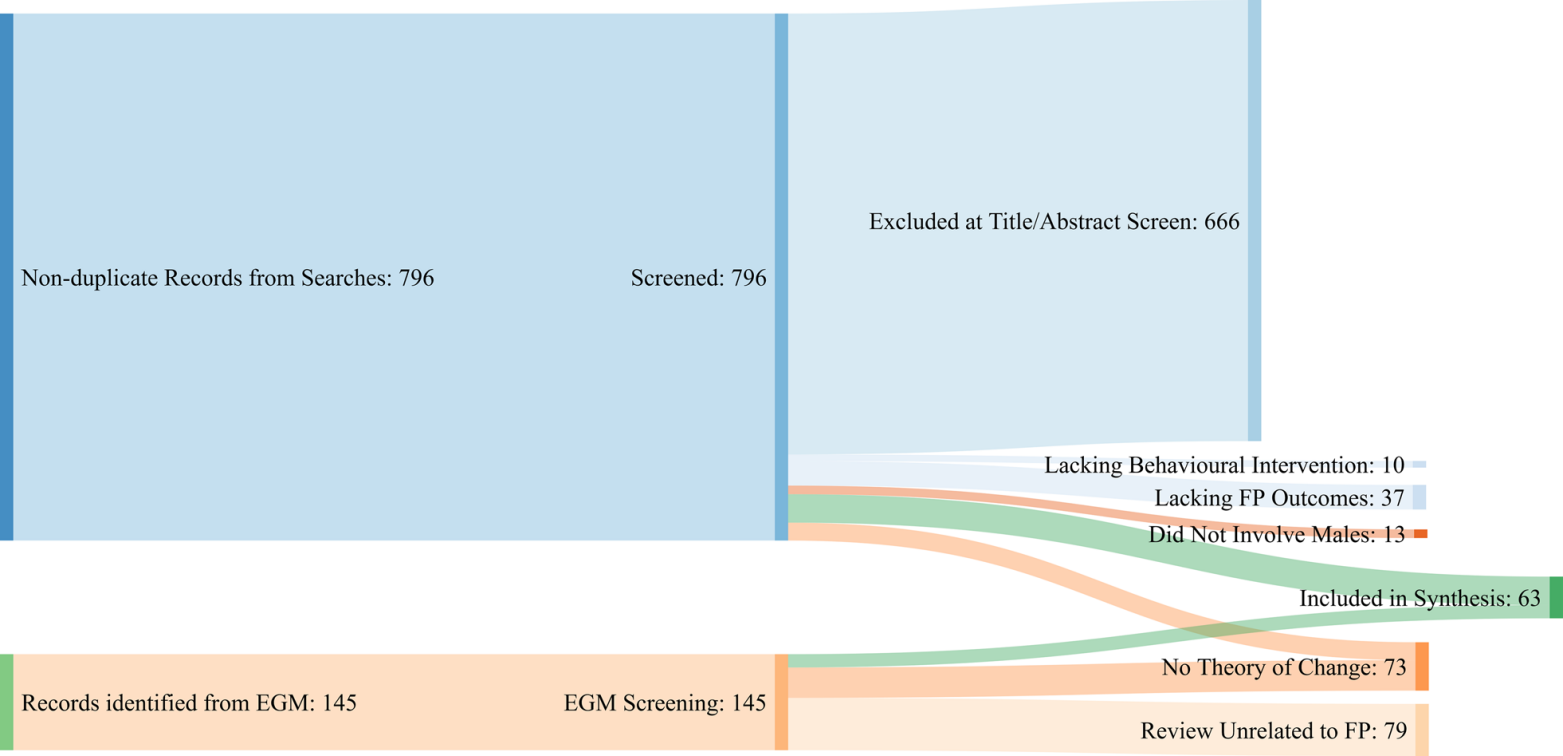
Inclusion and exclusion of records during screening

The search strategy returned 1066 records. Following removal of duplicates, 941 unique records were screened by title and abstract and 745 excluded. Of the remaining 196 records, 133 were excluded at full text screening leaving 63 records. See Figs. 1 and 2 to illustrate the survey flow and reasons for exclusion.

### Study and intervention characteristics

Of the 63 records included in this review, 21 were reports of individual pieces of research (reported in, for example, primary research articles or conference papers); 32 were systematic reviews of interventions; 8 were methodological reports (e.g. protocols) or technical papers related to an intervention or programme, and 2 were review commentaries on FP interventions and services. Several studies (*n* = 28) reported on interventions in multiple countries. More than half of all records (*n* = 41) contained information on interventions delivered in LMICs, most commonly South Africa (*n* = 13), India (*n* = 10), Zimbabwe (*n* = 8), Tanzania (*n* = 7), and Uganda (*n* = 6). Of the records detailing interventions conducted in high-income countries (HICs) the majority detailed interventions in the USA (*n* = 16), and UK (*n* = 4). Three systematic reviews [26–28], and one guidance document did not systematically detail the countries of implementation.

All cited interventions targeted outcomes related to FP behaviours. These were most frequently related to contraceptive uptake and use (most commonly condom use), and the modification of sexual behaviours, e.g. avoiding unprotected sex or abstaining from sex. Interventions also sought to promote FP service uptake and engagement. This included providing information about available services and enabling engagement with these. Interventions focused on adolescents more typically focused on outcomes such as abstinence, reduced unprotected sex, and reduced unintended pregnancy.

In terms of intervention strategy, the majority of cited interventions involved the provision of information within their components, particularly those targeting adolescent populations. These were typically in the form of sexual health curricula delivered in school settings, individual and group educational workshops, or the dissemination of materials (e.g. information, condoms). Other notable strategies for intervention in the same vein were the development and promotion of knowledge and skills around FP use through individual and group counselling, community outreach programmes, educational mass media, and peer communication. The use of such community-level strategies were notably more prevalent in LMICs [29–33].

Characteristics of included records are presented in Table 1.

**Table 1:** Study characteristics

Author (year)	Publication type	Country(ies)	Outcome(s)	Intervention strategies	Theory(ies) of change	Presentation
Agha (2019)	Primary research	Pakistan	Increased condom use	Social marketing	Fogg behaviour model	Narrative, figure
Arstide (2020)	Primary research	Tanzania	FP service uptake and use	Family planning Workshops for religious leaders	Social action theory	Narrative, figure
Asingwire (2019)	Review, impact evaluation	Uganda	FP service uptake and use, awareness, attitudes and self efficacy related to family planning	National family planning programme	Novel theory of change	Narrative, figure
Aventin (2015)	Intervention protocol	UK, Ireland	Reduced unintended pregnancy, increased contraceptive uptake and use	Video-based, educational	Theory of planned behaviour, and components of other sociological behaviour change theories	Narrative
Bailey (2010)	Systematic review	USA	Reduced unintended pregnancy, reduced HIV/STI prevalence	Interactive computer based interventions	Novel theory of change* Social cognitive theory; IMB skills model, Stages of change, Theory of planned behaviour	Narrative, figure, study characteristics table
Barker (2007)	Systematic review	Panama, Peru, Nicaragua, USA, South Africa, Costa Rica, UK, Australia, Canada, Turkey, Indonesia, Zimbabwe, India, Egypt, Pakistan, Jordan, Guinea, Bangladesh, Kenya, Ethiopia, Viet Nam, Nepal, Brazil, Argentina	Increase contraceptive use, increase FP communication, FP service uptake and use, reduced HIV/STI prevalence, reduced IPV	Group educations, community outreach, service provision	Gender transformative programming	Narrative
Berhane (2015)	Primary research	Ethiopia	FP service uptake and use, increased use of modern contraceptives	Service provision	Transtheoretical model of behaviour change	Narrative
Casey (2015)	Systematic review	Liberia	FP service uptake and use, increased condom use, reduced STI/HIV prevalence	School-based contraceptive intervention	Social change theory	Narrative
Chin (2012)	Systematic review	Not Specified	Contraceptive uptake, reduced unprotected sex, reduced pregnancy rate	Sexual health education, abstinence promotion	Novel theory of change*	Narrative, figure
Cornman (2007)	Primary research	India	Increased contraceptive use, reduced STI/HIV prevalence	Informational/educational workshops	IMB Skills Model	Narrative
Crankshaw (2012)	Primary research	South Africa	Increased contraceptive use, reduced STI/HIV prevalence	Theoretical model, behaviour change communication	Novel theory of change*, IMB skills model	Narrative, figure
Decat (2013)	Primary research, conference paper	Bolivia, Ecuador, Nicaragua	Increased sexual health information seeking, increased communication about sexuality, promotion of safe sex behaviours	Adolescent sexual health promotion campaign	Theory of planned behaviour, social cognitive theory	Narrative
Doubova (2016)	Protocol	Mexico	Increased contraceptive (condom) use, reduced sexual risk-taking behaviours, knowledge attitudes and self-efficacy related to condom use	Internet-based sexual health education	IMB skills model	Narrative
Downing (2011)	Systematic review	USA	Reduced unintended pregnancy, reduce sexual activity, increased HIV/STD knowledge	Parent–child and family interventions in increase knowledge and improve communication around adolescent sexual health	Social cognitive theory; diffusion of innovations model, theory of planned behaviour; eco-developmental theory; social learning theory; cognitive behaviour theory; relational ethics; social developmental theory	Study characteristics table
Fleming (2018)	Primary research	India	Gender equitable attitudes, family planning uptake, attitudes toward female contraceptive use	Gender equality and family planning counselling	theory of gender and power	Narrative, table of intervention content
Germ (2009)	Primary research	USA	Change in parenting practices, reduced adolescent sexual risk taking behaviours	Parental discipline and adolescent coping training	Social control theory	Narrative
Gottschalk (2014)	Systematic review	Mexico, Cameroon	Contracptive (condom) use	Educational workshops, sexual health peer-education	Social cognitive theory, theory of reasoned action, theory of planned behaviour	Study characteristics table
Guse (2012)	Systematic review	USA	Reduced unprotected sex, knowledge of HIV/STI and pregnancy prevention, sexual activity attitudes, refusal self-efficacy, sexual initiation/ debut, number of sexual partners, reduced sexual risk behaviour	Web and technology-based interventions	Social cognitive theory, theory of planned behaviour, theory of reasoned action, "motivational enhancement therapy", self-regulation theory, extended parallel process model, transtheoretical model, social influence models, theory of triadic influence	Narrative, Study Characteristics Table
Haberland (2015)	Systematic review	Zimbabwe, USA, Kenya, South Africa	Reduced unintended pregnancy, reduced STI infection, contraceptive uptake, condom use skills, reduce childbearing prevalence, more gender-equitable relationships, knowledge attitudes and skills around unprotected sex, Increased communication and negotiation skills, Improved sexual health knowledge	Informational videos, educational, workshops, peer-education	Novel theory of change, social learning theory, stages of change model, social cognitive theory, theory of planned behaviour, social development theory, theory of gender and power, socioecological model of behaviour change, social inoculation theory, cognitive behaviour theory, health belief model	Narrative, study characteristics table
Hobgen (2015)	Systematic review	USA, UK, Turkey	Reduced unintended pregnancy, reduced unprotected sex, knowledge and attitudes related to contraceptive use, carrying condoms, condom use improved sexual health, HIV/AIDS status, STD status,	Parent–child interventions to prevent unintended pregnancy, educational workshops and seminars, school- and university-based sexual education	Domains of sexual health model* Social cognitive theory, life skills theory	Narrative, figure
Jackson (2012)	Systematic review	South Africa, Namibia, USA	Prevent substance use and risky sexual behaviour, Improved knowledge of reproductive biology, reduced HIV/STI transmission, reduced drug use, better healthy development, reduced alcohol consumption, intra-relationship violence, communication skills	School-based, sex education curriculum	Life skills theory, social cognitive theory	Study characteristics table
Jennings (2014)	Primary research	USA	Preventing unintended pregnancy, reducing HIV/STI infection	School-based sex education programme	Social learning theory, health belief model	Narrative
Kilwein (2017)	Systematic review	USA	Reduced alcohol related unprotected sex, reduced alcohol in conjunction with sex, number of sexual partners	University-based, safe sex messaging, counselling, reminders	Motivational interviewing, personalised normative feedback	Narrative
Kraft (2014)	Systematic review	Egypt, Ethiopia, Bangladesh, Tanzania, South Africa, India, Pakistan, Nepal, Brazil, Malawi, Nigeria, Guatemala, El Salvador, Guinea, Ghana, Zimbabwe	Family planning use, reproductive behaviour and health outcomes	Couples counselling, community mobilisation. group meetings and workshops, educational activities, mass media campaigns	Gender equality continuum*	Narrative, figure
Kulathinal (2019)	Primary research	India	Enhancing knowledge of, and practices related to, reversible contraceptives	mHealth, mobile helpline, distribution of contraceptives	Novel theory of change	Narrative, figure
Levy (2019)	Systematic review	USA, Senegal, India, Egypt, Nepal, Vietnam, Ethiopia, Mexico, Brazil, Chile, Zimbabwe, Zambia, South Africa, Kenya, Uganda	Family planning, reduced IPV, nutrition, maternal and child health, hygiene, infectious disease	Community engagement, education and awareness raising, promoting economic stability, enabling physical environment changes	Gender transformative programming*	Narrative, figure
Lohan (2015)	Primary research	UK, Ireland	Reducing unintended adolescent pregnancy, contraceptive uptake	Video-based, school-based education and activities	Theory of planned behaviour	Narrative
Lopez (2009)	Systematic review	USA, UK, Tanzania	HIV/STD risk reduction, reduced unintended pregnancy, reduced unsafe sexual behaviours, influence knowledge, attitudes and norms around contraceptive use, school staff and parent knowledge, Increased self-efficacy and social support for contraceptive use, self-efficacy, intentions, behaviour planning, social and communication skills, positive gender norms and power	School-based sex education curriculum, risk assessment and health behaviour training, parental education, community and school linkage,	Social cognitive theory, theory of reasoned action information-motivation-behaviour skills, social influence theory social learning theory, cognitive behavioural theory, protection motivation theory	Narrative, study characteristics table
Lopez, Bernholc (2016)	Systematic review	USA, UK, Mexico, South Africa	Increased contraceptive use, reduced unintended pregnancy	School-based sex education curriculum	Social cognitive theory, theory of planned behaviour; social learning theory, health belief model, cognitive behavioural theory, I-change model social influence theory, and theory of triadic influence	Study characteristics table
Lopez, Grey (2016)	Systematic review	USA, Scotland, Guatemala, India, South Africa	Improve contraceptive use (uptake, continuation), reduced unintended pregnancy	School-based sex education, group-based education	Social cognitive theory, social learning theory, social influence theory, theory of planned behaviour, theory of triadic influence; theory of gender and power, information-motivation-behaviour skills model, motivational interviewing; transtheoretical model, cognitive behavioural theory health belief model, I-change model	Narrative
Lundgren (2019)	Primary research	Uganda	Improved sexual health and rights, perception of contraceptive use, greater gender equality, reduced gender based violence	Community mobilisation	Social Constructivist Perspective, Gender Theory (Butler, 1990)	Narrative
MacArthur (2018)	Systematic review	USA, South Africa	Reduced risky health behaviours, reduced adolescent sexual risk-taking behaviour	School-based, educational workshops, skills training workshops, computer-based counselling, conditional payments, home visits from health practitioners, family workshops, informational videos	Novel theory of change* Social learning theory, behavioural economics, cascading pathways model, cognitive behavioural theory, social learning theory, problem behaviour theory, social development theory, theory of triadic influence, life skills theory, theory of reasoned action framework, I-change model, social influence theory	Narrative, figure, study characteristics table
Mason (2016)	Systematic review	England, Scotland, Zimbabwe, Kenya, South Africa, Tanzania	Prevention of HIV/STI transmission, reduced unintended adolescent pregnancy, increased used of male condoms, delayed sexual initiation (debut), improved sexual behaviour knowledge and attitudes	School-based sex education, peer education	Novel theory of change*	Narrative, figure
Mirzazadeh (2018)	Systematic review	USA	STI/HIV prevention, adolescent sexual activity, condom use (at last intercourse, regularly)	School-based sex education curriculum, parental education	Natural opinion leader model, social learning theory, health belief model, social development model	Narrative, study characteristics table
Moreno (2014)	Systematic review	China, India, Peru, Russia, Zimbabwe	Reduced incidence of unprotected sex, reduced HIV/STI prevalence, reduced pregnancy incidence, improved behaviours knowledge and attitudes related to sex	Community mobilisation, community leader engagement, group-based education	Innovation diffusion theory, social learning theory	Narrative
Mukamuyango (2020)	Primary research	Rwanda	Family planning service use, long acting contraceptive (LARC) uptake, discussion of LARC	Couple-based family planning counselling, individual motivational interviewing	Motivational interviewing	Narrative
Munro (2019)	Primary research	USA	Contraception uptake, shared decision-making regarding contraceptive use, programme implementation feasibility	Service provider training, video resources and decision aids	Transtheoretical domains framework, COM-B Model	Narrative
Munro (2017)	Primary research (conference paper)	USA	Shared decision making in contraceptive uptake, intervention acceptability and feasibility	Not specified	Transtheoretical domains framework	Narrative
Mwaikambo (2011)	Systematic review	Honduras, Nicaragua, and Mexico, Tanzania, Botswanan, Philippines	Use of family planning services, knowledge and attitudes about family planning, discussions around family planning, intentions to use family planning, contraceptive use, unmet family planning need, reduced unintended pregnancies and abortion, total fertility rate	School-based sex education curriculum	Social learning theory, social cognitive theory	Study characteristics table
NCT, Wagman (2014)	Intervention protocol	Uganda	Reduced sexual risk behaviours, reduced non-marital partnerships, increased contraceptive use, reduced intimate partner violence, reduced HIV infection	Community mobilisation	Transtheoretical model (stages of change theory)	Narrative
Nguyen (2013)	Technical report	Bangladesh	Uptake of modern contraceptives, improved healthcare service provision, increased access to health services, skilled birth attendant, improved infant feeding behaviours	Community outreach, service provision	PerFORM framework, socio-ecological model	Narrative, figure
Poobalan (2009)	Systematic review	USA, UK, Canada	Reduced teen pregnancy, improved sexual health, reduced HIV/AIDS	School/community-based education, lectures, role-plays, games, skills workshops	Social learning theory, social cognitive theory, theory of planned behaviour, theory of reasoned action, health belief model, social inoculation theory	Narrative
Pretorius (2015)	Systematic review	South Africa, Uganda, Zimbabwe, Democratic Republic of Congo	Number of sexual partners, condom use, consistent condom use, communication about condom use, sexual activity, condom use skills, negotiation and communication skills around safe sex	Group-based counselling	Cognitive behaviour theory, social cognitive theory	Narrative
Rink (2016)	Primary research	USA	Reduced HIV/STI infection, reduced unintended pregnancy, improved relationship communication skills, condom use attitudes and self-efficacy, sexual decision making, sexual risk behaviours, relationship power	Peer-based outreach, peer-led education, and skills building sessions	Theory of reasoned action	Narrative
Rodríguez (2013)	Systematic review	Belize, Namibia	Improved knowledge, attitudes, and intentions to use condoms, Improved known access to condoms, Intention to use condoms, fewer sexual partners, delay of sexual debut	Sexual education sessions/curriculum	Framework for voluntary family planning programs that respect, protect, and fulfill human rights* Social learning theory, social cognitive theory	Narrative, figure
Schriver (2017)	Systematic review	Countries not specified All geographic regions represented	Reduced HIV/AIDS, reduced gender-based violence, healthy timing and spacing of pregnancy, adolescent and youth health, safe motherhood, infant and child health and nutrition, tuberculosis	Health promotion interventions with gender transformative programming	Novel theory of change, social learning theory, social cognitive theory, social behavioural change communication, theory of reasoned action, IMB skills model, diffusion of innovation theory	Narrative
Schuler (2015)	Primary research	Guatemala	Greater gender equitable attitudes, improved modern contraceptive knowledge, increased modern contraceptive use	Interactive workshops, individual and couple-based workshops	C-change social and behaviour change model, social ecological model	Narrative, figure
Shattuck (2011)	Primary research	Malawi	Improved contraceptive knowledge, contraceptive uptake	Community outreach and mentoring programme, peer-led outreach, education, and skills training	IMB skills model	Narrative
Shelus (2018)	Primary research	Rwanda	Increased contraceptive awareness, increased family planning uptake, intentions to use family planning	Community mobilisation, educational entertainment radio programming, community discussion groups	Social learning theory	Narrative
Steinfield (2018)	Primary research	Uganda	Improved understanding sexual power relations, promoting contraceptive use, promoting sexual health service use	Social marketing	Transformative gender justice framework	Narrative, figure
Sweat (2012)	Systematic review	Mozambique, India, South Africa, Cameroon, Zambia	Contraceptive (condom) uptake and use	Social marketing, mass media	Novel theory of change*	Narrative, Figure
Tékponon Jikuagou Project (2013)	Review commentary	East Timor, Ghana, Tanzania, India, Democratic Republic of Congo, Mali, Rwanda, Vietnam, Bangladesh	Family planning uptake and use, reduced HIV/STI prevalence, increased contraceptive use	Individual education, group education, service provision, mass media, community mobilisation	Socio-ecological model	Narrative, figure
Tolli (2012)	Systematic review	Italy, Germany, Italy, Greece	Contraceptive use, number of sexual partners, sexual health knowledge, relationship communication, contraceptive attitudes, condom use intentions	Peer-education, school-based sex education, sexual education with HIV prevention	Social learning theory, diffusion of innovation	Study characteristics table
Underhill (2008)	Systematic review	USA	HIV prevention, sexual abstinence, contraceptive use, sexual activity, delayed sexual debut, contraceptive/ STI knowledge	School-based sexual health education and promotion, community facility educational campaigns, after school or saturday school educational sessions, parent–child assigned homework	Social cognitive theory, social inoculation theory, social influence theory, theory of reasoned action, theory of planned behaviour, protection motivation theory, life skills theory, health belief model, self-efficacy theory, cognitive behaviour theory, IMB skills model	Narrative, study characteristics table
Underhill (2007)	Systematic review	USA	HIV prevention, sexual abstinence, unprotected sex, STI prevalence, pregnancy prevalence, condom use, delayed sexual initiation	School-based abstinence promotion, parent–child education and engagement	Novel theory of change, cognitive behaviour theory, social learning theory, social cognitive theory, theory of possible selves, social inoculation theory	Narrative
Underwood (2015)	Primary research	Jordan	Improved knowledge and more positive attitudes related to family planning and gender relations, more positive health messaging/preaching, greater exposure to positive FP-related messaging, increased likelihood of FP behaviour change related to messages	Faith-based promotion of contraceptive use	Carey communication model, ideation theory	Narrative
UNFPA (2014)	Inception report	Not specified	Universal access and capacity for family planning provision and uptake	Advocacy, service development, knowledge translation and dissemination, capacity development for FP service engagement	Novel theory of change	Narrative, figure
UNFPA (2012)	Business case	Zimbabwe	Provision and uptake of family planning services	Social marketing, mass media, community mobilisation and communication, subsisted contraceptive and FP services, improved service provision and integration	Novel theory of change	Narrative, figure
UNRWA (2018)	Service implementation report	Syria, Gaza, Jordan	Gender inclusive service provision and practice for refugees, male participation in preconception care, improved understanding of family planning and reproductive health, improved parenting outcomes	Educational campaign and workshops	Novel theory of change	Narrative, figure
USAID (2017)	Guideline document	USA, Bangladesh, Honduras, Ghana, Uganda, South Africa, Tanzania, Malawi, Namibia, Lesotho, Swaziland, Mozambique	Effective family planning service provision and engagement	Education, counselling, peer-educators, social media information dissemination and service promotion, community mobilisation, mass-media campaigns, social marketing/ tailored communication	Socio-ecological model, gender equality continuum	Narrative
Wakhisi (2011)	Systematic review	USA, UK	Increased parent–child communication about sex and contraception, increase behavioural self-control, improved knowledge of reproductive health, attitudes toward abstinence, remove barriers for abstinence, delayed sexual initiation/debut, increased condom use, self -efficacy to refuse unwanted sex, reduced unintended pregnancy, reduced unprotected sex	School-based sex education, skills training at family planning centres, sex education for school teachers and parents	Novel theory of change, social cognitive theory, social learning theory, cognitive behavioural theory, social influence theory, Carrera model, health belief model, theory of reasoned action, theory of planned behaviour	Study characteristics table
Webb (2016)	Systematic review	USA	Reduced prevalence of unplanned pregnancy, reduced unprotected sex, reduced STI prevalence	Motivational interviewing, health counselling sessions	Motivational interviewing, '5A’ framework for behavioural counselling, social cognitive theory	Study characteristics table
Zapata (2015)	Systematic review	Not specified	Reduced teen pregnancy, increased contraceptive use, use of more effective contraceptives, correct contraceptive use, contraceptive continuation, increased and repeat use of family planning services, increased knowledge, increased psychosocial determinants of contraceptive use	Healthcare provider contraceptive counselling, peer-led counselling	Novel theory of change*, motivational interviewing	Narrative, figure

*Theory of change applied to review synthesis

### Theories of change

Over half of records (*n* = 73, 55%) were excluded at the full text screening stage because they did not include detail on an intervention theory of change. Among included intervention studies and reviews, most provided a narrative description of a theory of change and how it was applied in the study (*n* = 56, 89%). Just under half of these also featured graphical or diagrammatic representations of theories of change accompanying a narrative summary (*n* = 21, 33%). Reviews of interventions were also found to report the underlying theory of change for included interventions when presenting the study characteristics (*n* = 17, 27%). While many records did describe the application of theory of behaviour change narratively, this was often limited and lacked granular detail on how the intervention components were built on the underpinning behaviour change theory.

Several studies reported that intervention theories of change were based on established behaviour change theory. The most frequently cited behaviour change theories on which interventions were based were Social Cognitive Theory [34], Social Learning Theory [8], the Theory of Planned Behaviour/Reasoned Action [7, 35], and the Information-Motivation-Behaviour [IMB] Skills Model [36]. A summary of the theories of change identified across records may be found in Box 1. Most often, the theoretical frameworks cited were centred on individual level factors or influences of behaviour.

A central tenet among the most popular intervention theories of change was integrating elements of improving knowledge and skills and promoting more positive social norms around FP and sexual health behaviours. These factors were encompassed in some way by the aforementioned most popular theories of behaviour change. This was also further exemplified by reviews that presented a conceptual theory of change applied to studying several interventions [37–43]. These reviews also typically drew on existing behaviour change theories, synthesising a general proposed theory of change and applying this to the context and content investigated.

While interventions were chiefly focused on individual-level factors, some cited interventions incorporated theories of change with environmental and structural features, for example citing the Social-Ecological Model [31, 44–46]. Moreover the C-Change Model cited by Schuler and colleagues [31] described encouraging behaviour change communication at multiple levels of influence (interpersonal, community, and environmental). Furthermore, records detailing more holistic implementations [e.g. 47] incorporated elements within their novel theory of change related to environmental factors, such as improved service provision, in addition to strategies for individual factors.

Some interventions were also reported to incorporate multiple theories of change concurrently. For example, Jennings and colleagues [48] report the evaluation of a peer-led sex education intervention with adolescents in the USA: the Teen PEP Model. This programme was described as adopting a ‘multi-theoretical approach’ that incorporated Social Learning Theory, the Health Belief Model, and principles of Youth Leadership Development. These theories were used in tandem to improve adolescent knowledge, skills, and ultimately behaviours to avoid unintended pregnancy and sexually transmitted infections (STIs).

We identified several examples of interventions and programmes that detailed a novel theory of change, developed in tailored way for a specific intervention. One example of this was applied by Kulathinal and colleagues [49]. This intervention drew on a theory of change developed through desk-based research that identified three key issues in contraceptive uptake: a lack of information, gender bias, and unavailability or inaccessibility of contraceptives. This process model was used to develop intervention targets and activities to address these directly. This novel theory of change also notably incorporated information provision and gender-aware strategies. Other authors described more tailored theories of change developed based on the needs of a particular population and prior evidence. For instance, the PerFORM Framework and I-Change Model [42, 50] were presented as novel theories of change based on previously published behaviour change theories.

A minority of cited interventions detailed integration of gender-aware theories. Some examples of these were the Theory of Gender and Power [51], Gender Theory [52], Transformative Gender Justice Framework [53], Gender Transformative Programming [54], and the Gender Equality Continuum [55]. Gender-aware theories were less widely cited and defined than more traditional theories of behaviour change. A key record identified in relation to the use of gender in interventions, however, was the review by Schriver and colleagues [28]. This examined the evaluation of 99 gender-aware and -transformative health promotion interventions as per the Interagency Gender Working Group definition of the Gender Equality Continuum. It found that interventions with a novel theory of change were more likely to incorporate aspects of gender-awareness and transformation. In contrast, our review examined theories of change as reported by authors and reviewers, rather than the reviewers applying gender theory in investigation of existing interventions, which may explain the divergence in findings.

Box 1 Summary of theories of change identified in review

**Table:** 

"5A" Framework for Behaviour Counselling Behavioural Economics Carey Communication Model Carrera Model Cascading Pathways Model C-Change Cognitive Behaviour Theory COM-B Model Diffusion of Innovations Eco-developmental Model Extended Parallel Process Model Framework for Voluntary Family Planning Programs that Respect, Protect, and Fulfil Human Rights Fogg Behaviour Model Gender Equality Continuum Gender Theory Gender Transformative Programming Health Belief Model I-Change Ideation Theory IMB Skills Model Innovation Diffusion Theory Life Skills Theory Motivational Enhancement Therapy Motivational Interviewing Natural Opinion Leader Model Novel Logic Models/Theories of Change	PerFORM Framework Personalised Normative Feedback Principles of Youth Leadership Development Problem Behaviour Theory Protection Motivation Theory Self-Efficacy Theory Self-Regulation Theory Social Development Theory Social Action Theory Social Behavioural Change Communication Social Change Theory (Attitudes, Skills, Self-Efficacy) Social Cognitive Theory Social Constructivist Perspective Social Control Theory Social Influence Theory Social Inoculation Theory Social Learning Theory Socio-Ecological Model Theory of Gender and Power Theory of Planned Behaviour Theory of Possible Selves Theory of Reasoned Action Theory of Triadic Influence Transformative Gender Justice Framework Transtheoretical Domains Framework Transtheoretical (Stages of Change) Model Youth Leadership Development

## Discussion

Kurt Lewin [56, p. 129] claimed that “nothing is as practical as a good theory”, because, he argued, good practice is underpinned by rigorous understanding of the dynamics that influence it. With his adage in mind, we aimed to scope the range of behaviour change theories applied in FP interventions involving men and boys. The findings provide an overview of contemporary practices and reporting on which future programme developers and evaluators might draw to inform their designs.

We found that FP programmes involving men and boys employ a range of behaviour change theories. Among those most frequently cited within intervention studies and reviews were Social Cognitive Theory [34], Social Learning Theory [8], the Theory of Planned Behaviour/Reasoned Action [7, 35], and the IMB Skills Model [36]. These findings echo those of previous reviews of theory-based interventions to encourage contraceptive use among women [14, 15] and, therefore highlight a potential commonality in FP intervention designs regardless of participant gender.

Notwithstanding these commonalities, we also found that a diverse range of theoretical approaches were applied, with one or more of over 50 different novel or existing theories mentioned in the included studies. This likely reflects diversity in programme aims and objectives, target population, and contextual factors such as the implementation setting, which can, and should, influence the choice of theoretical approach [57]. Such a range of potential theoretical options may be perplexing for programme developers and suggests the need for intervention development guidelines to direct planners in this regard. A recent systematic review [58] reports a wide range of published health intervention development frameworks, some of which incorporate guidance on utilising behaviour change theory [59, 60].

Our review findings also highlight considerable heterogeneity in reporting of theories of change. While most records described theories of change using narrative methods, and to a lesser extent figures or logic models detailing processes, there was considerable variation in the level of detail provided in these. This suggests that the design and theories of change underpinning interventions should be substantively and consistently reported. The use of reporting frameworks for intervention design and components, e.g. the TIDiER guidelines [61], may benefit future evidence synthesis and programme development [13].

We have also found that, while cited in relation to some interventions, gender norms and gender structures remain under addressed in FP programmes involving men and boys. Only 14 percent (n = 9) of the included studies reported that their theory of change was informed by theories of gender. This supports findings of a previous review of health interventions with gender-theory integration, which reported considerable heterogeneity in approaches applied. Given the importance of promoting gender equality in relation to sexual and reproductive health and rights as a catalyst for change [37], the inclusion of gender-aware and gender-transformative theory and applications will be an important consideration for future programme design. Further, examination of the potential impact of doing so would be a worthy consideration for future evaluation research.

It should be considered that the publications reviewed here may have lacked exhaustive detail of intervention programming and theory in their reporting, even when these were present. It is possible that programmes are underpinned by behaviour change theory in their development and implementation, but that this goes unreported or underreported [28]. For instance, word limits imposed on academic publications might restrict the provision of information on theoretical underpinning. Moreover, even when interventions are successful, it may be difficult to determine what theoretical components and strategies have effected this, because this level of detail and evaluation is not often available to readers [14]. We therefore, emphasise the need for detail to be provided relating to theoretical underpinnings and expected causal mechanisms of behaviour change prospectively and evaluation of these to better understand what strategies are truly effective and how these affect changes.

Equally, trends in intervention design and reporting might be heavily influenced by the priorities in funding provided [38]. For example, the results of this review noted the frequent use of community-based interventions to promote contraceptive use in LMICs and of interventions promoting condom use which affects family planning and STI/HIV outcomes. It is possible that those programmes seeking to address both of these health issues are more likely to receive funding for implementation and subsequently published. There is, therefore, a need both for funders of intervention programmes to prompt or explicitly require implementers to detail any design and theoretical processes underpinning FP programmes, and for specific evaluation of contraceptive use intentions (i.e. for purposes of FP or SRHR) resulting from these interventions.

Finally, it should be noted that a large contributor to the exclusion of records was the absence or limited reporting of intervention theory of change. This accounted for more than half (55%) of the exclusions at the full-text screening stage. The implication of this is that theories of change used by FP interventions with men and boys may remain poorly understood or overlooked by readers and may fail to promote potentially valuable intervention strategies. This further highlights the need improve reporting of intervention design and use of theories of change in this area or to provide this information in supplementary material [62]. Given the importance of well-founded theory of change in intervention success [6] there remains a need for clear description and evaluation of this in intervention research. We therefore recommended that authors and publishers should make use of standardised frameworks (e.g. CONSORT, TIDieR) in reporting of intervention evaluation and design [63]. Intervention developers should not shy away from proposing and testing theories and reporting the process and results unambiguously to promote the development of theory and practice.

### Implications

These findings highlight the diversity of behaviour change theory featured in FP programmes involving men and boys, and the diversity of reporting. We provide a useful overview for intervention programmers hoping to learn from the current state of theory of change in current FP programmes. We also highlight a call to action for future development and adaptations of interventions to unambiguously detail the use of theories of change and to not shy away from evaluation causal mechanisms of programmes. The implications for researchers are likewise to report on intervention theories of change sufficiently and consistently in evaluations and reviews, and to facilitate investigation of how theoretical components effect behaviour change.

### Limitations

This review included a broad range of evidence from intervention studies, reviews, and methodological protocol publications. While this provided a wide range of data from which to draw conclusions, it is possible there exists some overlap in the reporting of interventions, i.e. programmes reported multiple times across several reviews. As such, this review does not make claims about the absolute prevalence of intervention characteristics and application of theory. Rather the results of this review are indicative of practices more generally in this area.

The results of this review are descriptive of only programme design in relation to theory of change and do not capture the entire range of potential influencing factors that might influence intervention fidelity and effectiveness. It must, therefore, be acknowledged that numerous factors may exist that contribute to the success of an intervention not described by this review. The intended theory of change of an intervention may be considered only one facet of behaviour change that exists within the complexities of wider context and unmeasured extraneous factors that also affect the target behaviour [64].

The final limitation to acknowledge is the restricted nature of this review in relation to data acquisition (i.e. limiting searching to title, abstract, and keywords). This approach allowed for the timely gathering and synthesis of data regarding a specific research question, but limits understanding of the full range of theories of change. Likewise, it should be highlighted that these results detail the theories and frameworks applied by interventions involving men and boys, making no claims regarding their efficacy to influence behaviour change for individuals or groups. While evidence tentatively indicates that the results of rapid reviews coincide with full systematic reviews [16], these results and analysis should be interpreted with appropriate caution, acknowledging that these can be used to inform an overview of theories of change rather providing than an exhaustive list.

## Conclusion

This review provides an overview of contemporary practices and reporting with regards to the use of theories of behaviour change in FP programmes involving men and boys. The large number of screened records excluded due to a lack of information on theory of change and variability in reporting highlights a need for programmers and authors to make clear the underpinnings of their programmes. Given the importance of well-founded theory affecting change, this information is essential for future reviewers and programmers to make decisions on what constitutes good practice in FP interventions with men and boys. The presented evidence synthesis provides an overview of the intended mechanisms of change within current FP interventions, and is a call to action for authors to rigorously detail the use and application of theory in future programmes and for journal editors to allow them to do so.

## Data Availability

The datasets used analysed during the current study are available from the corresponding author on reasonable request.

## Appendix 1: Search terms

The search terms used were informed by the primary goal of this review; namely, to identify the underpinning theoretical framework of FP interventions with men and boys. An initial list of terms was reviewed by three members of the research team for face validity. The terms they agreed were then piloted in each target database and refined prior to implementation.

Terms used for the Family Planning and Males concepts were informed by those used by the previous systematic review of SRHR undertaken by members of the research team from which initial evidence was obtained [21]. The terms used for the Framework concept were adapted from those recommended by Booth and Carroll [65] and Rehfuess an colleagues [66].

**Table:**

1. Family planning	2. Males	3. Intervention type	4. Intervention design	5. Framework
“Family planning” OR contraception OR “birth spacing” OR “child spacing” OR “unplanned pregnancy” OR “unintended pregnancy” OR “unwanted pregnancy” OR abortion OR *fertility	Men OR man OR male OR males OR boy OR boys OR masculin* OR father* OR husband OR partner	Intervention* OR program* OR trial* OR random*	behav* OR educat* OR psycho* OR social	framework* OR model* OR ((theor* adj2 (change OR behav*)) OR concept* OR diagram* OR figure* OR construct OR principle*

Searches were limited to article Title, Abstract, and Keywords as appropriate across databases. The search strategy specified “1 AND 2 AND 3 AND 4 AND 5”, limiting results to publications from 2007 to present.

## Appendix 2: References for included studies

1. Agha S, Tollefson D, Paul S, Green D, Babigumira JB: Use of the Fogg Behavior Model to Assess the Impact of a Social Marketing Campaign on Condom Use in Pakistan. *J Health Commun* 2019, 24(3):284–292.
2. Aristide C, Mwakisole A, Mwakisole N, Emmanuel M, Laizer E, Kihunrwa A, Downs D, Wamoyi J, Downs J: Design and pilot testing of a church-based intervention to address interpersonal and intrapersonal barriers to uptake of family planning in rural Tanzania: a qualitative implementation study. *BMJ Sex Reprod Health* 2020, 46(3):226–233.
3. Asingwire N, Muhangi D, Kyomuhendo S, Leight J: Impact evaluation of youth-friendly family planning services in Uganda. In*.* New Delhi, India: International Initiative for Impact Evaluation; 2019.
4. Aventin A, Lohan M, O'Halloran P, Henderson M: Design and development of a film-based intervention about teenage men and unintended pregnancy: applying the Medical Research Council framework in practice. *Eval Program Plann* 2015, 49:19–30.
5. Bailey JV, Murray E, Rait G, Mercer CH, Morris RW, Peacock R, Cassell J, Nazareth I: Interactive computer-based interventions for sexual health promotion. *Cochrane Database Syst Rev* 2010(9):CD006483.
6. Barker G, Ricardo C, Nascimento M: Engaging Men and Boys in Changing Gender-Based Inequity in Health: Evidence from Programme Interventions. In*.* Geneva, Switzerland: World Health Organisation & Promundo; 2007.
7. Berhane A, Biadgilign S, Berhane A, Memiah P: Male involvement in family planning program in Northern Ethiopia: an application of the Transtheoretical model. *Patient Educ Couns* 2015, 98(4):469–475.
8. Casey SE: Evaluations of reproductive health programs in humanitarian settings: a systematic review. *Confl Health* 2015, 9(1):S1.
9. Chin HB, Sipe TA, Elder R, Mercer SL, Chattopadhyay SK, Jacob V, Wethington HR, Kirby D, Elliston DB, Griffith M et al.: The effectiveness of group-based comprehensive risk-reduction and abstinence education interventions to prevent or reduce the risk of adolescent pregnancy, human immunodeficiency virus, and sexually transmitted infections: two systematic reviews for the Guide to Community Preventive Services. *Am J Prev Med* 2012, 42(3):272–294.
10. Cornman DH, Schmiege SJ, Bryan A, Benziger TJ, Fisher JD: An information-motivation-behavioral skills (IMB) model-based HIV prevention intervention for truck drivers in India. *Soc Sci Med* 2007, 64(8):1572–1584.
11. Crankshaw TL, Matthews LT, Giddy J, Kaida A, Ware NC, Smit JA, Bangsberg DR: A conceptual framework for understanding HIV risk behavior in the context of supporting fertility goals among HIV-serodiscordant couples. *Reproductive Health Matters* 2012, 20:50–60.
12. Decat P, De Meyer S, Jaruseviciene L, Ibarra M, Medina J, Auquilla N, Hagens A: Community embedded reproductive health interventions for adolescents in Latin America: development of a complex intervention. *European journal of contraception & reproductive health care* 2013, 18:S98‐S99.
13. Doubova SV, Infante-Castañeda C, Pérez-Cuevas R: Internet-based educational intervention to prevent risky sexual behaviors in Mexican adolescents: study protocol. *BMC Public Health* 2016, 16(1):1–8.
14. Downing J, Jones L, Bates G, Sumnall H, Bellis MA: A systematic review of parent and family-based intervention effectiveness on sexual outcomes in young people. *Health Education Research* 2011, 26(5):808–833.
15. Fleming PJ, Silverman J, Ghule M, Ritter J, Battala M, Velhal G, Nair S, Dasgupta A, Donta B, Saggurti N et al.: Can a Gender Equity and Family Planning Intervention for Men Change Their Gender Ideology? Results from the CHARM Intervention in Rural India. *Stud Fam Plann* 2018, 49(1):41–56.
16. German M: An experimental test of parental influences on risky sexual behaviors among mexican-origin adolescents. *Dissertation Abstracts International: Section B: The Sciences and Engineering* 2009, 70(5-B):3170.
17. Gottschalk LB, Ortayli N: Interventions to improve adolescents' contraceptive behaviors in low- and middle-income countries: a review of the evidence base. *Contraception* 2014, 90(3):211–225.
18. Guse K, Levine D, Martins S, Lira A, Gaarde J, Westmorland W, Gilliam M: Interventions Using New Digital Media to Improve Adolescent Sexual Health: A Systematic Review. *Journal of Adolescent Health* 2012, 51(6):535–543.
19. Haberland NA: The Case for Addressing Gender and Power in Sexuality And HIV Education: A Comprehensive Review Of Evaluation Studies. *International Perspectives on Sexual and Reproductive Health* 2015, 41(1):31-U106.
20. Hogben M, Ford J, Becasen JS, Brown KF: A Systematic Review of Sexual Health Interventions for Adults: Narrative Evidence. *Journal of Sex Research* 2015, 52(4):444–469.
21. Jackson C, Geddes R, Haw S, Frank J: Interventions to prevent substance use and risky sexual behaviour in young people: a systematic review. *Addiction* 2012, 107(4):733–747.
22. Jennings J, Howard S, Perotte C: Effects of a school-based sexuality education program on peer educators: The Teen PEP model. *Health Education Research* 2014, 29(2):319–329.
23. Assessing the Impact of an Intervention to Prevent Intimate Partner Violence and HIV in Uganda [https://clinicaltrials.gov/show/NCT02050763].
24. Kilwein TM, Kern SM, Looby A: Interventions for Alcohol-Related Risky Sexual Behaviors Among College Students: A Systematic Review. *Psychol Addict Behav* 2017, 31(8):944–950.
25. Kraft JM, Wilkins KG, Morales GJ, Widyono M, Middlestadt SE: An Evidence Review of Gender-Integrated Interventions in Reproductive and Maternal-Child Health. *Journal of Health Communication* 2014, 19(sup1):122–141.
26. Kulathinal S, Joseph B, Saavala M: Mobile Helpline and Reversible Contraception: lessons From a Controlled Before-and-After Study in Rural India. *JMIR mhealth and uhealth* 2019, 7(7):e12672.
27. Levy JK, Darmstadt GL, Ashby C, Quandt M, Halsey E, Nagar A, Greene ME: Characteristics of successful programmes targeting gender inequality and restrictive gender norms for the health and wellbeing of children, adolescents, and young adults: a systematic review. *The Lancet Global Health* 2020, 8(2):e225-e236.
28. Lohan M, Aventin A, Oliffe JL, Han CS, Bottorff JL: Knowledge translation in men's health research: Development and delivery of content for use online. *Journal of Medical Internet Research* 2015, 17(1):e31.
29. Lopez LM, Bernholc A, Chen M, Tolley EE: School‐based interventions for improving contraceptive use in adolescents. *Cochrane Database of Systematic Reviews* 2016(6).
30. Lopez LM, Grey TW, Chen M, Tolley EE, Stockton LL: Theory‐based interventions for contraception. *Cochrane Database of Systematic Reviews* 2016(11).
31. Lopez LM, Tolley EE, Grimes DA, Chen-Mok M: Theory-based strategies for improving contraceptive use: a systematic review. *Contraception* 2009, 79(6):411–417.
32. Lundgren R, Chantelois H, Burgess S, Kågesten AE, Oregede S, Kerner B: Processing gender: lived experiences of reproducing and transforming gender norms over the life course of young people in Northern Uganda. *Culture, Health & Sexuality* 2019, 21(4):387–403.
33. MacArthur G, Caldwell DM, Redmore J, Watkins SH, Kipping R, White J, Chittleborough C, Langford R, Er V, Lingam R et al.: Individual‐, family‐, and school‐level interventions targeting multiple risk behaviours in young people. *Cochrane Database of Systematic Reviews* 2018(10).
34. Mason‐Jones AJ, Sinclair D, Mathews C, Kagee A, Hillman A, Lombard C: School‐based interventions for preventing HIV, sexually transmitted infections, and pregnancy in adolescents. *Cochrane Database of Systematic Reviews* 2016(11).
35. Mirzazadeh A, Biggs MA, Viitanen A, Horvath H, Wang LY, Dunville R, Barrios LC, Kahn JG, Marseille E: Do School-Based Programs Prevent HIV and Other Sexually Transmitted Infections in Adolescents? A Systematic Review and Meta-analysis. *Prevention Science* 2018, 19(4):490–506.
36. Moreno R, Nababan HY, Ota E, Wariki WMV, Ezoe S, Gilmour S, Shibuya K: Structural and community‐level interventions for increasing condom use to prevent the transmission of HIV and other sexually transmitted infections. *Cochrane Database of Systematic Reviews* 2014(7):CD003363.
37. Mukamuyango J, Ingabire R, Parker R, Nyombayire J, Easter SR, Wall KM, Tichacek A, Nyirazinyoye L, Kaslow N, Allen S et al.: Motivational interviewing to promote long-acting reversible contraception among Rwandan couples wishing to prevent or delay pregnancy. *American Journal of Obstetrics & Gynecology* 2020, 222(4):S919.e911-S919.e912.
38. Munro S, Manski R, Donnelly KZ, Agusti D, Stevens G, Banach M, Boardman MB, Brady P, Bradt CC, Foster T et al.: Investigation of factors influencing the implementation of two shared decision-making interventions in contraceptive care: a qualitative interview study among clinical and administrative staff. *Implementation science* 2019, 14(1):95.
39. Munro S, Manski R, Donnelly KZ, Agusti D, Stevens G, Banach M, Boardman MB, Brady P, Colon Bradt C, Foster T et al.: Right for me: feasibility and acceptability of two shared decision-making interventions for contraceptive methods. *Contraception* 2017, 96(4):289‐.
40. Mwaikambo L, Speizer IS, Schurmann A, Morgan G, Fikree F: What works in family planning interventions: A systematic review of the evidence. *Stud Fam Plann* 2013, 42(2):67–82.
41. Nguyen NK, Rahman S: Behaviour Change Communication Strategy for the Urban Primary Health Care Services Delivery Project. In: *Technical Assistance Consultant’s Report.* Edited by Bank AD; 2013.
42. Poobalan AS, Pitchforth E, Imamura M, Tucker JS, Philip K, Spratt J, Mandava L, van Teijlingen E: Characteristics of effective interventions in improving young people's sexual health: a review of reviews. *Sex Education* 2009, 9(3):319–336.
43. Pretorius L, Gibbs A, Crankshaw T, Willan S: Interventions targeting sexual and reproductive health and rights outcomes of young people living with HIV: a comprehensive review of current interventions from sub-Saharan Africa. *Global health action* 2015, 8:28,454.
44. Rink E, Ricker A, FourStar K, Anastario M: Unzip the truth: Results from the Fort Peck Men's Sexual Health Intervention and Evaluation Study. *American Journal of Sexuality Education* 2016, 11(4):306–330.
45. Rodriguez M, Harris S, Willson K, Hardee K: Voluntary Family Planning Programs That Respect, Protect, And Fulfill Human Rights: A Systematic Review of Evidence. In*.* Washington, D.C.: Engender Health; 2013.
46. Schriver B, Mandal M, Muralidharan A, Nwosu A, Dayal R, Das M, Fehringer J: Gender counts: A systematic review of evaluations of gender-integrated health interventions in low- and middle-income countries. *Global Public Health* 2017, 12(11):1335–1350.
47. Schuler SR, Nanda G, Ramirez LF, Chen M: Interactive workshops to promote gender equity and family planning in rural communities of Guatemala: Results of a community randomized study. *Journal of Biosocial Science* 2015, 47(5):667–686.
48. Shattuck D, Kerner B, Gilles K, Hartmann M, Ng'ombe T, Guest G: Encouraging Contraceptive Uptake by Motivating Men to Communicate About Family Planning: The Malawi Male Motivator Project. *American Journal of Public Health* 2011, 101(6):1089–1095.
49. Shelus V, VanEnk L, Giuffrida M, Jansen S, Connolly S, Mukabatsinda M, Jah F, Ndahindwa V, Shattuck D: Understanding your body matters: Effects of an entertainment-education serial radio drama on fertility awareness in Rwanda. *Journal of Health Communication* 2018, 23(8):761–772.
50. Steinfield LA, Coleman CA, Tuncay Zayer L, Ourahmoune N, Hein W: Power logics of consumers’ gendered (in)justices: reading reproductive health interventions through the transformative gender justice framework. *Consumption Markets & Culture* 2018, 22(4):406–429.
51. Sweat MD, Denison J, Kennedy C, Tedrow V, O'Reilly K: Effects of condom social marketing on condom use in developing countries: a systematic review and metaanalysis, 1990–2010. *Bull World Health Organ* 2012, 90(8):613–622.
52. Tékponon Jikuagou Project: From Family Planning to Fatherhood: Analysis of Recent Male Involvement Initiatives and Scale-Up Potential. In*.* Washington, D.C.: Institute for Reproductive Health, Georgetown University; 2013.
53. Tolli MV: Effectiveness of peer education interventions for HIV prevention, adolescent pregnancy prevention and sexual health promotion for young people: a systematic review of European studies. *Health Education Research* 2012, 27(5):904–913.
54. Underhill K, Montgomery P, Operario D: Abstinence‐plus programs for HIV infection prevention in high‐income countries. *Cochrane Database of Systematic Reviews* 2008(1).
55. Underhill K, Operario D, Montgomery P: Abstinence‐only programs for HIV infection prevention in high‐income countries. *Cochrane Database of Systematic Reviews* 2007(4).
56. Underwood CR, Kamhawi SS: Friday sermons, family planning and gender equity attitudes and actions: evidence from Jordan. *Journal of Public Health* 2015, 37(4):641–648.
57. UNFPA: Sexual and reproductive health and HIV prevention in Zimbabwe: Business Case. In*.* New York, N.Y.: United Nations Populations Fund; 2012.
58. UNFPA: Evaluation of The UNFPA Support to Family Planning 2008–2013. In*.* New York, N.Y.: United Nations Populations Fund; 2014.
59. UNRWA: UNRWA Gender Equality Strategy 2016–2021: Annual Implementation Report January-December 2018. In*.* Jerusalem, Israel United Nations Relief and Works Agency; 2018.
60. USAID: Guide for Promoting Sexual and Reproductive Health Products And Services For Men. In*.* Baltimore, M. D.: Johns Hopkins Center for Communication Programs; 2017.
61. Wakhisi AS, Allotey P, Dhillon N, Reidpath DD: The effectiveness of social marketing in reduction of teenage pregnancies: A review of studies in developed countries. *Social Marketing Quarterly* 2011, 17(1):56–90.
62. Webb MJ, Kauer SD, Ozer EM, Haller DM, Sanci LA: Does screening for and intervening with multiple health compromising behaviours and mental health disorders amongst young people attending primary care improve health outcomes? A systematic review. *BMC Fam Pract* 2016, 17:12.
63. Zapata LB, Tregear SJ, Curtis KM, Tiller M, Pazol K, Mautone-Smith N, Gavin LE: Impact of Contraceptive Counseling in Clinical Settings: A Systematic Review. *American Journal of Preventive Medicine* 2015, 49(2):S31-S45.
